# Understanding access to healthcare among Indigenous peoples: A comparative analysis of biomedical and postcolonial perspectives

**DOI:** 10.1111/nin.12237

**Published:** 2018-03-25

**Authors:** Tara Horrill, Diana E McMillan, Annette S H Schultz, Genevieve Thompson

**Affiliations:** ^1^ Rady Faculty of Health Sciences College of Nursing University of Manitoba Winnipeg MB Canada; ^2^ Health Sciences Centre Winnipeg MB Canada

**Keywords:** critical analysis, critical theory, health services, health services accessibility, healthcare access, Indigenous health, Indigenous peoples, postcolonial theory

## Abstract

As nursing professionals, we believe access to healthcare is fundamental to health and that it is a determinant of health. Therefore, evidence suggesting access to healthcare is problematic for many Indigenous peoples is concerning. While biomedical perspectives underlie our current understanding of access, considering alternate perspectives could expand our awareness of and ability to address this issue. In this paper, we critique how access to healthcare is understood through a biomedical lens, how a postcolonial theoretical lens can extend that understanding, and the subsequent implications this alternative view raises for the nursing profession. Drawing on peer‐reviewed published and gray literature concerning healthcare access and Indigenous peoples to inform this critique, we focus on the underlying theoretical lens shaping our current understanding of access. A postcolonial analysis provides a way of understanding healthcare as a social space and social relationship, presenting a unique perspective on access to healthcare. The novelty of this finding is of particular importance for the profession of nursing, as we are well situated to influence these social aspects, improving access to healthcare services broadly, and among Indigenous peoples specifically.

## 1 INTRODUCTION

Access to healthcare is widely acknowledged as a social determinant of health (CSDH, 2008). In Canada, reasonable access to healthcare services without financial or other barriers (Canada Health Act, 1985) and the equitable distribution of healthcare services (referring to the fair distribution of healthcare services based on need) are desired goals of the healthcare system (Cameron, del Pilar Carmargo Plazas, Santos Salas, Bourque Bearskin, & Hungler, 2014). However, evidence indicates that access is neither equal nor equitable among all Canadians, and Indigenous peoples in Canada tend to have more difficulty accessing healthcare services than non‐Indigenous Canadians (CIHI, 2004; NCCAH, 2011; Peiris, Brown, & Cass, 2008). While healthcare access issues are well documented among Indigenous peoples in Canada, our understanding of access is dominated by a biomedical perspective, providing minimal insight into the social, political, and historical influences on access to healthcare. A lack of attention to these influences is common in studies of the health inequities and disparities experienced among Indigenous peoples, and hinders efforts to redress health inequities, including those related to access to healthcare (Browne, Smye, & Varcoe, 2005; Peiris et al., 2008; Prior, 2007). Alternatively, adopting a postcolonial perspective facilitates an expanded understanding of inequities in access to healthcare among Indigenous peoples by attending to the social, political, and historical influences.

In this paper, we provide a critical comparative analysis of how access to healthcare is conceptualized and understood through two theoretical perspectives: biomedical and postcolonial. This comparative analysis was the result of doctoral work completed by the lead author (TH), in which the objective was to critique our common understanding of ‘access to healthcare’ and compare that to an alternative theoretical conceptualization. In addition, we were interested in how the expanded understanding of access to healthcare might guide the profession of nursing, and what role nursing might play in redressing access inequities experienced among Indigenous peoples. We begin with a discussion of biomedical perspectives and how these inform our collective understanding of healthcare access. We then define postcolonial perspectives, contrasting how healthcare access is understood through this lens. We discuss key implications to nursing knowledge and practice that emerge with a postcolonial understanding of access to healthcare. As a means to focus the argument**,** we have applied this analysis to the experiences of Indigenous peoples within Canada, drawing largely on the Canadian experience to enable understanding of the complex and dynamic contextual influences on Indigenous health, and access to healthcare. The Canadian experience is an illustration of ideas that are relevant to other healthcare contexts, along with an understanding that health inequities and disparities among Indigenous peoples are a global concern (Gracey & King, 2009).

### 1.1 Biomedical theoretical perspectives

Biomedical theory, or the medical model, refers to a group of theories rather than a distinct, single theoretical perspective and includes theories from medicine, biology, epidemiology, and other disciplines (McEwen & Wills, 2014). Epistemologically, biomedical theories subscribe to objectivism, the philosophical view that meaning exists apart from knowledge, and knowledge awaits discovery (Crotty, 1998). Biomedical theories are rooted in positivism and empiricism and thus emphasize observation, reduction, verification, prediction, control, and objectivity in the discovery of knowledge (McEwen & Wills, 2014). Empiricism is strongly linked with the development of liberal political philosophy (Browne, 2001), and neoliberalism, liberalism's modern equivalent, exercises significant influence in biomedical theories (McGibbon & Hallstrom, 2012). A central tenet of liberalism is that of individualism, in which the individual is ‘abstracted from their social, economic, political or historical context’ (Browne, 2001; p. 121). Individualism ushered in the individual as the principal scientific unit of analysis, fundamentally linking liberalism with empiricism; individualism continues its influence within positivist and postpositivist traditions, including biomedicine (Browne, 2001). Within this purview, health is considered the absence of disease, taking a reductionistic view of disease at the individual level, as the result of a single, linear cause (Yuill, Crinson, & Duncan, 2011).

Biomedical research is responsible for many medical discoveries and has played a significant role in the discovery and advancement of treatments for both acute and chronic diseases. However, within biomedical theories, the social origins of disease that extend beyond individual choice or responsibility are not considered (Thompson, 2016). Biomedical theories of health and healthcare dominate the healthcare system in Canada and have had a significant influence in nursing theory, research, and practice (McEwen & Wills, 2014; Risjord, 2010).

### 1.2 Biomedical perspectives on access to healthcare

#### 1.2.1 Conceptualization

Given the dominance of biomedical perspectives in healthcare, access to and inequities in access to healthcare are primarily known through a biomedical lens. From a biomedical perspective, access to healthcare is conceptualized as dependent on the physical accessibility of services. Physical accessibility is related to geographical distance, availability of services, and healthcare providers, or financial ability to overcome geographical distance to access services**.** Given the influence of neoliberalism and its central tenet of individualism within biomedicine, access tends to be viewed at the level of the *individual*. Individual awareness of healthcare services as well as individual choice and responsibility to access those services is important components of access to healthcare as defined by biomedical perspectives. Underlying individual choice and responsibility to access services is the assumption that healthcare services are equally accessible to all and that individuals have the freedom to choose to access healthcare services—nods to the tenets of egalitarianism and individual freedom within liberalism.

#### 1.2.2 Barriers to access

Access to healthcare is frequently understood through the description and understanding of what access is *not* (barriers to access). Health Canada (PHAC, 2008) acknowledges barriers to accessing healthcare for Indigenous peoples in Canada and cites the availability of services and healthcare providers to on‐reserve First Nations as barriers. The availability and retention of healthcare providers (Lavoie, Kaufert, Browne, & O'Neil, 2016), long wait lists (Health Council of Canada, 2013; PHAC, 2008), and limited access to screening and preventative services (NCCAH, 2011; PHAC, 2008) are repeatedly noted as barriers to accessing healthcare. The Health Council of Canada's (2013) report on healthcare renewal focused solely on wait times as a measure of healthcare access. Geographical distance and the corresponding costs of transportation for those living in rural or remote areas impede access to healthcare (CPAC, 2013). These factors can all be categorized as contributing to physical inaccessibility of services (Lavoie et al., 2016).

Other factors are viewed as contributors to inaccessibility of health services. Lower levels of education are associated with poorer health, and this trend is reported among Indigenous peoples in Canada (CIHI, 2004); one underlying argument is that lower education results in a lack of awareness regarding early detection and preventative care, which is a barrier to accessing healthcare (NCCAH, 2011). Financial barriers to accessing healthcare may be created when the costs of services are not covered (PHAC, 2008). A complex and fragmented healthcare system for Indigenous peoples in Canada, complicated by differences in health coverage between First Nations, Inuit and Métis Peoples, and differences in services provided among and between provinces, creates further barriers to accessing healthcare (National Collaborating Centre for Aboriginal Health (NCCAH), 2011).

#### 1.2.3 Solutions to improve access

Conceptualizing access as dependent on the physical availability of healthcare services and healthcare providers, as well as individual responsibility for awareness of services and individual decision to access those services, informs strategies for improving access. Based on this rationalization, when healthcare is conceptualized as a ‘service’, increasing service provision and thereby increasing physical access would lead to improved healthcare access (Tang, Browne, Mussell, Smye, & Rodney, 2015). In addition, strategies to improve awareness of healthcare services and encourage decisions to access those services, such as educational and marketing campaigns, as well as increasing funding for medical transportation, are cited (CPAC, 2013; PHAC, 2008).

### 1.3 Postcolonial theoretical perspectives

The theoretical underpinnings of a postcolonial perspective are rooted in critical social theory and postmodern social theory. The emergence of this standpoint was in response to the dominance of a scientific philosophy in which legitimated empirically observed or logically deduced knowledge was valued (Campbell & Bunting, 1999; McEwen & Wills, 2014). Postcolonial theories subscribe to a constructionist epistemology, whereby meaning is constructed through social interaction (Crotty, 1998), and along with other critical social theories, are rooted in analyzing power dynamics (Reimer‐Kirkham, Baumbusch, Schultz, & Anderson, 2007). Postcolonial scholarship in particular is concerned with themes related to race and racism, and issues related to power imbalances, specifically those with historical roots such as colonial relations (Anderson et al., 2009). Postcolonial theories draw our attention to the historical, economic, cultural, and social contexts of health and healthcare (Reimer‐Kirkham et al., 2007), providing an approach to critically analyze colonial experiences and their ongoing manifestations in the health and wellness of Indigenous peoples (Browne et al., 2005). Moreover, research informed by postcolonial theories studies the intersectional relationships between race, poverty, gender, and other factors, which are seen as having synergistic or compounding negative effects on health (Browne et al., 2011).

Postcolonial perspectives have advanced our understanding of race, which has been a debated concept among and between biomedical and social science communities (Braun, 2002; Witzig, 1996). Scientific evidence from the fields of genetic and evolutionary biology has not supported biological origins of race; rather race is argued to be a social construction (Braun, 2002; Glenn, 2000; Witzig, 1996). Race therefore is a concept that has been used to construct and organize dominant and subordinate relationships (Reimer‐Kirkham & Anderson, 2002). *Racialization* is a process of labeling a group collectively based on ‘presumed biological, physical, or genetic differences’ and attributing social and cultural differences to race (Browne et al., 2005; p. 21). Similarly, *othering* describes the process by which ‘identity is assigned, human existence is categorized, people are characterized according to certain criteria (such as worldview or similar anthropological construct), and experiences are homogenized’ (Reimer‐Kirkham & Anderson, 2002, p. 6). Racialization and othering both result in generalizations and assumptions and are powerful mechanisms to create and maintain dominant–subordinate relationships (Reimer‐Kirkham & Anderson, 2002). Conceptualizing race as based on genetic ‘difference’ rather than as a social construct profoundly impacts how health research questions are framed, and whether the impact of social inequities or racial discrimination on health is considered (Braun, 2002).

It should be noted that postcolonial theory as discussed here arises from Western epistemologies rather than Indigenous epistemologies. Indeed, Browne et al. (2005) note the distinction between postcolonial theoretical perspectives and postcolonial Indigenous knowledge and summarize critique of postcolonial theoretical perspectives. Postcolonial discourse has the potential to essentialize the experiences of the colonized, who are diverse populations and groups of people (Browne et al., 2005). Attending to unique differences in experience cannot be overstated. Postcolonial perspectives have a tendency to create binary categories of colonizer and colonized, which ignore shifting social categories and intersecting oppressions, and risk perpetuating the ‘very power relations we seek to dismantle’ (Browne et al., 2005; p. 25). Despite critique, postcolonial theoretical perspectives can provide a much needed critical perspective and have been used increasingly by several Canadian nurse scholars conducting Indigenous health research (Browne et al., 2011; MacDonald, 2013).

### 1.4 Postcolonial perspectives on access to healthcare

#### 1.4.1 Conceptualization

Examining access to healthcare through a postcolonial lens is a means to understand the impact of race and power dynamics, as well as historical, political, and social influences on access. Through a postcolonial perspective, healthcare is understood as a form of social relationship, rather than strictly as a service (Tang et al., 2015). Healthcare spaces are conceptualized as both clinical spaces and social spaces. Tang et al. (2015) define social spaces as the ‘invisible and contested space in which people from different social positions negotiate access to power and resources’ (p. 710). Accordingly, access to healthcare includes not only the *availability* of services and providers, but also the *delivery* of services at the point of care, a significant component of which is the social relationship between provider and patient (Cameron et al., 2014; McGibbon, Etowa, & McPherson, 2008). In addition, analysis of access to healthcare through a postcolonial lens reveals structural disadvantages that shape opportunities, conditions, and the health of Indigenous peoples (Browne, 2012). Accessing healthcare extends beyond physical accessibility to equally consider the influence of contextual factors, and the social, historical, and political barriers that must be navigated by patients.

#### 1.4.2 Barriers to access

Power dynamics and imbalances are particularly noteworthy within a postcolonial perspective and theorized to be at the root of inequities in healthcare (Anderson et al., 2009). Previous negative experiences with healthcare services and/or healthcare providers among Indigenous peoples in Canada have repeatedly been found to create a barrier to accessing healthcare. Overcoming the fear of being judged on the basis of race or social class leads to delays in seeking care (Browne et al., 2011; Cameron et al., 2014; Denison, Varcoe, & Browne, 2014). Racism at the individual level, both overt and tacit (Browne et al., 2011; Denison et al., 2014; McGibbon et al., 2008), and discrimination, intimidation, and harassment (Cameron et al., 2014) impede the development of trusting relationships with healthcare providers and hinder access to healthcare. Historical trauma, referring to the effects (direct and intergenerational) of colonization and residential schools on Indigenous peoples in Canada, contributes significantly to difficulties in accessing healthcare (MacDonald, 2013; Wakewich, Wood, Davey, Laframboise, & Zehbe, 2016). The undercurrent of power differentials inherent in the colonization of Indigenous peoples in Canada has resulted in distrust of healthcare provided by the provincial and federal governments, which is an obstacle to accessing healthcare among Indigenous peoples (Wakewich et al., 2016).

Although healthcare services may be physically available and accessible, the *appropriate* and necessary care is not always received, often the result of assumptions and stereotyping on the part of healthcare providers (Cameron et al., 2014; Lavoie et al., 2016). Assumptions are the consequence of the ‘othering’ and ‘us versus them’ mentality that frequently occurs in healthcare, which must be overcome by those placed in the ‘other’ category, to access needed healthcare (Browne, 2007). While physical care may (or may not) be accessed and received, the disengagement of healthcare providers as a result of othering mentalities reduces the *quality* of care received (Browne, 2007). Othering has been conceptualized as social distancing or creating social space between categories, impeding access to healthcare (Tang et al., 2015).

A postcolonial perspective allows us to view healthcare through a political lens, drawing attention to political and power dynamics at play within healthcare (Reimer‐Kirkham & Anderson, 2002). The patchwork of healthcare policy and funding that is the current reality for Indigenous peoples in Canada creates difficulties accessing necessary healthcare services (Lavoie, 2013). In Canada, jurisdiction over the provision of healthcare for some Indigenous peoples has been contested for decades between federal and provincial levels of government, resulting in jurisdictional uncertainties and disputes that are ongoing. Healthcare services may be provided by the federal government, provincial government, or local Indigenous community depending on multiple factors. Consequently, many Indigenous peoples must cross these jurisdictional ‘borders’ to access appropriate healthcare—borders which are neat in theory, but messy and ambiguous in reality. Rarely is this a smooth or seamless process (Lavoie et al., 2015). The degree of perceived jurisdictional responsibility and the associated fiscal responsibility can ultimately determine whether healthcare services are provided to a particular patient or are not (Cook, 2003). These decisions are often made on a case‐by‐case basis, with the deciding factor being cost containment (Lavoie et al., 2015). Consequently, Indigenous peoples are left with feelings of frustration, distrust, arbitrariness, and healthcare needs that remain unmet, with little recourse (Lavoie et al., 2015). These policies create barriers to accessing healthcare, perpetuate dominant–subordinate relationships, and are continually shaped by racializing discourse (Anderson et al., 2009).

#### 1.4.3 Solutions to improve access

Conceptualizing healthcare as a form of social relationship and conceptualizing access as dependent upon social, historical, and political contextual factors reveal radically different strategies to improving access. Improving access to healthcare, then, targets social relationships and social spaces, aiming to reduce social distance (Tang et al., 2015) and create spaces for healthcare that are safe, inviting, and socially accepting (Browne et al., 2011; Cameron et al., 2014). Intentionally developing such environments fosters agency and a sense of worthiness in seeking healthcare services (Browne et al., 2016). In addition, cohesive health strategies and policies spanning all levels of government and inclusive of Indigenous peoples are urgently needed to address policies impeding access.

Addressing historical influences resulting in poor access to healthcare will require confronting our colonial past along with ongoing current colonial relations**,** recognizing that ‘colonizing thinking and actions permeate the nursing profession’ (McGibbon, Mulaudzi, Didham, Barton, & Sochan, 2014, p. 186). Sustained attention and investment must be given to decolonizing nursing education and practice. ‘Decolonization’ is defined as a process rather than an outcome; with this understanding, value is given to Indigenous voices and epistemologies, and the influence of colonial and neocolonial ideologies, practices, and processes is actively exposed and resisted (McGibbon et al., 2014; Swadener & Mutua, 2008). Moreover, a decolonizing process develops a consistent ‘counter‐narrative’ and involves ‘critical examination and dismantling of individual and systemic assumptions and power relationships’ (Smylie, 2011, p. 183). We discuss the application of cultural safety to nursing, a concept further discussed below, as one example of a decolonizing strategy with the potential to foster critical examination of power relations, assumptions, and colonial ideologies pervasive in nursing practice (McGibbon et al., 2014).

## 2 DISCUSSION

Our analysis comparing conceptualizations of access to healthcare, specifically among Indigenous peoples, sheds light on the limitations of drawing on a singular perspective to understand how access to healthcare is problematic. A postcolonial perspective shifts the responsibility away from the individual and toward the contextual influences and structural disadvantages encountered when accessing healthcare. Drawing on postcolonial‐informed conceptualizations of access *in addition to* biomedical understandings broadens the scope of solutions to improve access to healthcare among Indigenous peoples (See Table 1: Summary of Perspectives on Access to Healthcare). Notably, the framework for understanding healthcare as a social space and social relationship provided by a postcolonial analysis is of particular relevance to the profession of nursing. The awareness of the social aspects of access to healthcare is an important consideration for nurses given how we are situated within healthcare. Nurses are well positioned to influence and improve access to healthcare services broadly and among Indigenous peoples in particular. Specific implications for nursing practice and nursing knowledge are addressed below.

**Table 1:** Biomedical and postcolonial perspectives and implications on access to healthcare

	Biomedical	Postcolonial
Conceptualization	Access as an individual responsibility A‐contextual	Access as a social responsibility, and form of social relationship Healthcare spaces as a social spaces Social, historical, and political contexts important
Barriers to Access	Geographical location Availability of services (primary care, screening, and preventative care Availability/retention of healthcare providers Financial (ability to pay for non‐insured care)	*Social* Previous negative experiences in healthcare (racism, discrimination, fear of judgment) → delays in seeking care, discourages trust in healthcare providers Language barriers Ongoing colonial relations within healthcare system and institutions reflected in ‘othering’ mentalities *Historical* Legacy of colonization leading to distrust in government‐provided healthcare Historical trauma leading to delays and difficulties accessing care *Policy* Variation in healthcare benefits depending on location of residency; patchwork policy Federal/provincial jurisdictional ambiguities
Solutions	Increase services provided Increase healthcare providers Increase awareness of available healthcare services Increase funding for medical transportation	Create socially accepting, safe, and inviting spaces to reduce social distance Investment in decolonizing education, research, and healthcare practices. Cultural safety as one decolonizing strategy Coordinate policy between levels of government

### 2.1 Healthcare as service or relationship? Nursing implications

In light of the persistent health inequities experienced by Indigenous peoples in Canada and worldwide, including differences in access to healthcare, nursing scholars have questioned our uncritical reliance on biomedical perspectives, challenged us to unmask assumptions, and questioned the roots of nursing's clinical knowledge (Anderson et al., 2009). More recently, nursing scholars have acknowledged the need for sustained attention to the structural determinants of health—the ‘causes of the causes’—in nursing knowledge and practice (McGibbon et al., 2014). Decolonizing processes, which shift emphasis away from the dominance of biomedical assumptions and the ‘colonial amnesia that constructs and maintains poor health status’, are urgently needed (McGibbon et al., 2014, p. 182). The hegemonic positioning of biomedical perspectives in healthcare and our subsequent failure to acknowledge the influence of the historical, political, and social contexts and inherent power dynamics result in an incomplete understanding of how many issues, including access to healthcare, are shaped for Indigenous peoples (Browne et al., 2005; Vukic, Gregory, & Martin‐Meisner, 2012). Like the hamster continually running around the wheel, strategies to reduce health inequities and improve access to healthcare have continually relied on biomedical understandings, which, on their own, are ineffective and hinder progress (Browne et al., 2005). Postcolonial‐informed perspectives demand that we consider the structural determinants of health, focusing our attention on issues of relationships, power, race, and racialization, and how these affect health and access to healthcare among Indigenous peoples (Browne et al., 2005).

Conceptualizing access to healthcare as based on physical accessibility results in solutions targeting increasing the number of services and healthcare providers and corresponding funding, both of which are important in the context of Indigenous health, but largely exclude nursing from the solution. However, if access to healthcare is dependent upon contextual factors located within our social relationships, then nursing, as a profession, has a significant role to play in improving access. Nurses are challenged to practice in ways that create socially safe spaces, disrupting the racializing discourses prevalent in healthcare settings today. Recognizing, acknowledging, and talking about widespread experiences of racism, intimidation, harassment, distrust, and fear of judgment are a starting place. Understanding the social influences on access to healthcare positions nurses in all areas of practice to improve access to healthcare, be it from the bedside or the boardroom. As for nurse scholars, we must strive for generating knowledge, informed by multiple ways of knowing, to understand the ways in which socioeconomic factors, history, and policy have intersected to profoundly affect Indigenous health.

### 2.2 Postcolonial perspectives informing nursing practice: linking nurses’ positionality and access to healthcare

Pervasive in healthcare and nursing discourse is the belief that race, culture, or cultural characteristics are the reason certain groups experience health, social, or economic problems (Browne, 2012). These racialized ideologies, prevalent in healthcare settings today, construct Indigenous peoples as the source of their own problems and poor health status (Anderson et al., 2009; Sherwood, 2013). Qualitative research among nurses indicates a propensity to overlook and ‘bracket out’ the historical, socioeconomic, and political conditions underlying poor health and other problems (Browne, 2012; p. 168). Contrary to egalitarian discourses in nursing that purport to treat all patients equally (Browne, 2012), a growing body of evidence suggests that racism, discrimination, and fear of judgment are commonly experienced by Indigenous peoples accessing healthcare, often at the hands of nurses. It is critical that we disrupt racializing practices in nursing, which attribute differences in socioeconomic and health status to race or culture, rather than to colonial practices and structural disadvantage (Browne et al., 2005). Racialized explanations of health result in healthcare services and providers that are unresponsive to individual needs and unsafe (Smith, Varcoe, & Edwards, 2005).

Cultural safety has increasingly been taken up in nursing over the past decades as a strategy to prompt critical analysis of prevailing racializing discourses and power imbalances within healthcare (Browne, 2012). Cultural safety, a concept developed by Maōri nurse Irihapeti Ramsden, draws on postcolonial theoretical concepts to recognize that the current health status and socioeconomic status of Indigenous peoples stem from a colonial history (Ramsden, 2015). Adopting a cultural safety approach within practice does not require nurses to become experts in other cultures, but rather focuses on developing and improving self‐awareness. Nurturing culturally safe practices occurs through critical self‐reflection, questioning the roots of one's assumptions and values, reflecting upon one's sociocultural positioning and power, and shifting power from the nurse to the patient (Brascoupé & Waters, 2009; Browne, 2012; Papps, 2015; Reimer‐Kirkham et al., 2007).

A cultural safety lens draws attention to the way access to healthcare is shaped by power imbalances (Denison et al., 2014). Postcolonial perspectives on access to healthcare and a culturally safe approach shift the focus from the ‘cultural characteristics’ of an individual or group, toward the culture of *healthcare itself* as part of the problem of access to healthcare. Previous negative experiences, including racism and discrimination, and a history of colonization result in difficulties developing trusting relationships, are disempowering, and create barriers to accessing healthcare. Moreover, what nurses may fail to recognize is the perception by our patients, accurate or otherwise, that *we* are the gatekeepers to the healthcare system (Richardson, Yarwood, & Richardson, 2017). Thus, the cultural safety imperative to critically reflect on our positionality in relation to our patients is brought to the forefront. A cultural safety lens requires us as nurses to consider our own assumptions, positionality, and culture in relation to our nursing practice, and how these assumptions create or perpetuate poor access to healthcare.

Cultural safety has been identified as a critical factor in healing and reconciliation, and nurses working within this framework can begin to develop ‘relationships based on acceptance, trust and safety’ (Brascoupé & Waters, 2009, p. 9). Thus, practicing within a cultural safety approach is indeed a *nursing* strategy to improving access to healthcare. Moreover, cultural safety can be an entry point to decolonizing nursing by exposing and resisting colonial ideologies and practices through challenging values and assumptions held within nursing, and constructing a counter‐narrative that de‐centers the voice of the nurse while privileging Indigenous voices (McGibbon et al., 2014). Increasingly, cultural safety training is available for healthcare providers in both workshop and online formats. The San'yas Indigenous Cultural Safety Training program, a pioneering example of cultural safety training developed by the Provincial Health Services Authority of British Columbia's Aboriginal Health Program, combines online training with skilled facilitators to guide participants through training, and training specific for those in healthcare fields (http://www.sanyas.ca). Similar training programs have been developed elsewhere in Canada (Ontario and Manitoba), but at present, cultural safety training is not mandatory for most healthcare providers.

### 2.3 Postcolonial perspectives informing nursing knowledge: context matters

Creating the knowledge base needed to inform action on health inequities requires a shift in research agendas. Knowledge based only on ‘objective’ research findings stripped of context is problematic, resulting in ‘incomplete epistemologies’ (Reimer‐Kirkham et al., 2007). In no way can biomedical knowledge be discarded, but rather it must be incorporated and built upon, integrating critical, contextual knowledge with biomedical and clinical knowledge (Anderson et al., 2009). Contextual evidence and knowledge, attending to the social, historical, and political contexts of health, are key to disrupting racialized assumptions; the ‘nature of the evidence needed is that which makes visible the *social* pathways that lead to health disparities’ (Reimer‐Kirkham et al., 2007, p. 32).

Evidence regarding contextual influences gained through postcolonial scholarship adds depth and breadth to existing knowledge and provides insights regarding root causes underlying inequities in health and access to healthcare, which focus our attention on the human suffering that has occurred as a result (Anderson, 2004). This wide‐angle perspective is key to exposing relationships between historical, social, and political contexts that lead to and perpetuate poor health and access to healthcare. While generalizable knowledge is often prioritized in research, the importance of contextual knowledge to nursing cannot be understated: ‘[it] can shed light on the complexity of the intersectionalities among problems such as poverty and suffering, and people's inability to access appropriate healthcare, and thus provides the basis for planning and implementing equitable care at local, national, and global levels’ (Anderson et al., 2009, p. 289).

Chinn and Kramer (2008) propose this type of knowledge as ‘emancipatory knowing’ and argue that emancipatory knowledge development makes structural and social change possible. Emancipatory knowing is the human ability to notice social injustice and inequity, but also to critically consider why these injustices are perpetuated or remain unnoticed, and what social and structural changes must be made to right these wrongs (Chinn & Kramer, 2008). Creating this knowledge is critical to enacting social justice, arguably a social and moral responsibility within nursing (Reimer‐Kirkham & Anderson, 2002). A small body of literature exploring access to healthcare for Indigenous perspectives, guided by postcolonial and other critical theories, sheds light on where change is needed—not only in the availability of healthcare services and providers, but also in creating spaces that are socially safe and accepting, fostering the development of trusting relationships with healthcare providers and designing healthcare services and policies that attend to the intersecting historical, socioeconomic, and political conditions experienced by Indigenous peoples in Canada. Research and inquiry guided by postcolonial and other critical theories must continue to develop the evidence base necessary to effect these changes.

## 3 CONCLUSION

The inequities in healthcare access and poor health experienced by Indigenous peoples in Canada and worldwide are well known and documented, however, rarely has relevant evidence translated into improved health. The root causes of inequitable access to healthcare are complex and can be better addressed when understood within the social, historical, and political contexts. Understandings of access to healthcare from a biomedical perspective and the expectation that biomedical solutions alone can address barriers to access are insufficient and will not be effective in addressing these barriers (Gracey & King, 2009; Peiris et al., 2008). Postcolonial theoretical perspectives draw attention to the context surrounding inequities in access to healthcare, providing a more effective and compelling framework for understanding ‘how health, healing, and human suffering are woven into the fabric of the socio‐historical‐political context’ (Browne et al., 2005, p. 19).

As nurses, we can no longer be complicit in perpetuating colonial structures and relationships that undermine Indigenous peoples health and access to healthcare. As we have argued, a postcolonial analysis uniquely frames our understanding of healthcare as a social space and social relationship. By situating access within a social domain, the role nurses can and ought to play in addressing access inequities becomes clear. Incorporating critical self‐reflection and integrating cultural safety approaches into nursing practice and furthering the development of contextual knowledge gained through postcolonial‐informed and other critical inquiry are key to addressing inequities in access to healthcare among Indigenous peoples both within and beyond our Canadian borders (Anderson et al., 2009). We, as humans and as nurses, have this capacity woven into our being: that which ‘gives rise to a realization that there is something wrong with the way things are, and that it is possible to change for the better’ (Chinn & Kramer, 2008, p. 79).
